# SNP Discovery Using a Pangenome: Has the Single Reference Approach Become Obsolete?

**DOI:** 10.3390/biology6010021

**Published:** 2017-03-11

**Authors:** Bhavna Hurgobin, David Edwards

**Affiliations:** 1 School of Agriculture and Food Sciences, University of Queensland, St. Lucia 4072, QLD, Australia; b.hurgobin@uq.edu.au; 2 School of Biological Sciences and Institute of Agriculture, University of Western Australia, Perth 6009, WA, Australia

**Keywords:** pangenome, single nucleotide polymorphism, SNP discovery, copy number variation, presence absence variation, gene, assembly, genetic diversity, core genome, variable genome

## Abstract

Increasing evidence suggests that a single individual is insufficient to capture the genetic diversity within a species due to gene presence absence variation. In order to understand the extent to which genomic variation occurs in a species, the construction of its pangenome is necessary. The pangenome represents the complete set of genes of a species; it is composed of core genes, which are present in all individuals, and variable genes, which are present only in some individuals. Aside from variations at the gene level, single nucleotide polymorphisms (SNPs) are also an important form of genetic variation. The advent of next-generation sequencing (NGS) coupled with the heritability of SNPs make them ideal markers for genetic analysis of human, animal, and microbial data. SNPs have also been extensively used in crop genetics for association mapping, quantitative trait loci (QTL) analysis, analysis of genetic diversity, and phylogenetic analysis. This review focuses on the use of pangenomes for SNP discovery. It highlights the advantages of using a pangenome rather than a single reference for this purpose. This review also demonstrates how extra information not captured in a single reference alone can be used to provide additional support for linking genotypic data to phenotypic data.

## 1. The Pangenome Concept

**Figure 1:**
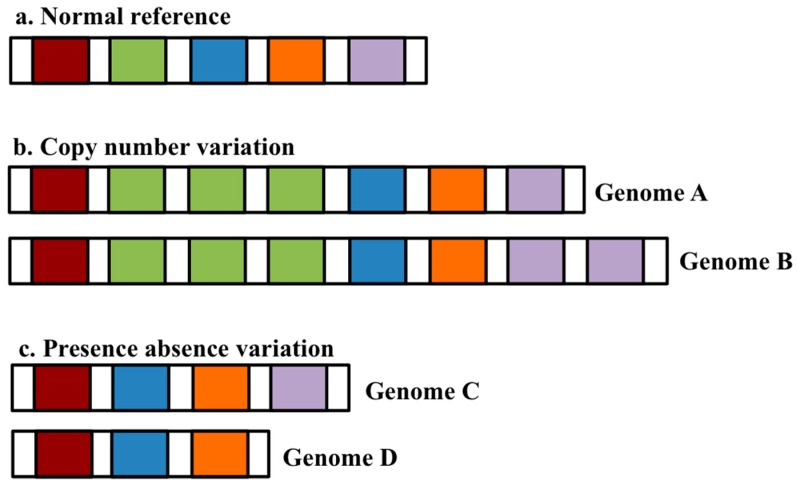
Figure illustrating a normal reference with the correct number of genes represented by coloured blocks (**a**), copy number variations (**b**) and presence absence variations (**c**).

It has become clear now that a single reference genome is not sufficient to fully represent the entire genetic diversity of a given species. This is due to the presence of structural variation in the form of copy number variants (CNVs) and presence/absence variants (PAVs), which alter the total amount of genetic information that is present within the individuals of the species [1]. CNVs are sequences, which occur in a different number of copies between individuals [2]. PAVs are sequences that are present in some individuals but absent in others; they represent an extreme form of CNV, where the sequence is completely missing from one or more individuals [1] (Figure 1). Therefore, in order to obtain the complete genomic content of any given species, its pangenome has to be constructed.

The concept of the pangenome was introduced in 2005 by Tettelin et al. [3], who produced the first ever pangenome for the bacterial species, *Streptococcus agalactiae*. This has led to a series of similar studies in other micro-organisms [4,5,6,7] as well as higher organisms including maize [8,9], soybean [10,11], rice [12,13], and Brassicas [14,15]. The pangenome can be thought of as the full complement of genes in a given species. It consists of the core genes, which are present in all individuals of the species, and variable/accessory/dispensable genes, which are present in some but not all individuals. The variable genes can further be divided into genes that are uniquely present, and genes that are present in two or more individuals [3,16]. The pangenome can either be closed/restricted or open. In the latter model, there appears to be no finite number of genes in the species, i.e., each newly added individual will add new genes to the pangenome. However, in the former model, the gene pool appears to be limited, i.e., once a certain number of individuals have been analysed, the addition of new individuals will not contribute to the expansion of the pangenome.

There are a number of factors that can determine how successful a pangenome study will be; these include the quality of the reference assembly, its annotation, and the selection of appropriate individuals [17]. The quality of the assembly in terms of its size, completeness, and fragmentation level will have a great impact on the quality of the annotation. The majority of genome assemblies produced to date have made use of short read technology for assembly purposes. However, this has prevented repetitive sequences from being resolved during the assembly process. This has resulted in highly fragmented assemblies, which are represented by a large number of contigs, and the positions of the repeat sequences remain unknown [18]. A fragmented assembly can give rise to a large number of partial genes, or even predict a smaller number of genes than what would exist. The latter case can affect downstream analyses such as functional annotation and gene presence/absence detection, among others. The same goes for incomplete assemblies, which can under-predict the total number of genes. Several metrics have been proposed to assess the level of completeness of genome assemblies, and these include, but are not limited to, the total length of the assembly, the N50, the number of contigs that span more than 500 bp [18], the number of core eukaryotic genes present as measured by Core Eukaryotic Genes Mapping Approach (CEGMA) [19], and more recently Benchmarking Universal Single-Copy Orthologs (BUSCO) [20]. Furthermore, the selection of appropriate individuals is essential to a successful pangenome study. The use of a small number of closely related individuals can significantly underestimate the pangenome size, so to get more realistic estimates, individuals that are as diverse as possible should be used [17]. Aside from choosing the most appropriate individuals, the number of individuals to include in the pangenome study is another aspect to consider [16]. This is not straightforward, but modeling of the pangenome expansion and core genome reduction can help answer this question. Several mathematical models based on the power law have been used for this purpose; these include a reduced model (y = A × B) and a complete model (y = A × B + C), where x is the number of genomes, y is the number of genes (pangenome or core), A is the multiplicative constant, B is the rate of decay, and C is the asymptotic number of minimum core genes [3,16,21].

**Figure 2:**
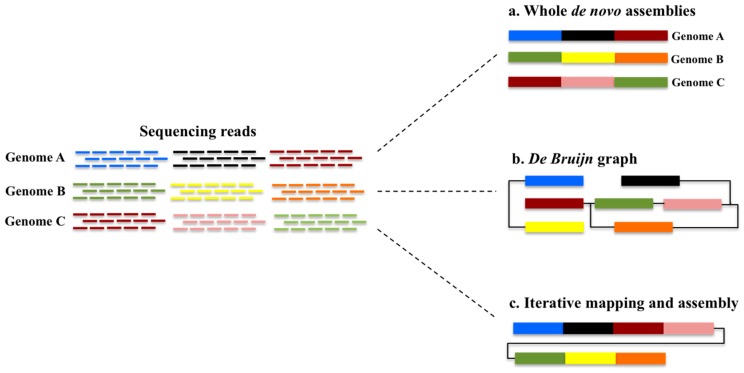
Different approaches to pangenome assembly. Three genomes (A, B and C) are shown and together they constitute a pangenome. Each genome consists of genomic segments that are marked by the same colour if present in multiple genomes. In the whole *de novo* assembly approach, the three genomes are assembled individually (**a**); In the *de Bruijn* graph approach, the genomes are broken down into segments and the relationships between segments can be traced back to the edges the graph (**b**); In the iterative mapping and assembly approach, a single genome is used as the basis and reads from other genomes are sequentially mapped and assembled, creating a non-redundant pangenome (**c**).

Several methods have been employed for assembling pangenomes; these include whole *de novo* genome assemblies, *k-mer* based approaches, and iterative mapping and assembly approaches. These are illustrated in Figure 2.

The whole de novo genome assembly approach is used when individuals have been sequenced to a high enough coverage that would allow them to be assembled individually [17]. In this way, the genomes can be aligned to each other in order to identify regions that are conserved or shared, and by extension identifying regions that display CNV. However, this would occur at the expense of requiring extensive data and computational resources, which may not always be available. In the *k-mer*-based approach, each sequence is broken down into shorter segments of length *k*. The relationship between these segments or *k-mer*s can be represented as edges of the graph where each *k-mer* is a node, and overlapping nodes are connected by an edge. The graph can include many edges, which connect to the same nodes, thereby forming loops. A genome can be reconstructed from *k-mer*s following relationships between the nodes. When more than one genome is present, additional information about the origin of the node has to be taken into consideration. This is achieved by colouring the nodes, with nodes originating from a given sample being assigned to a particular colour so that they can easily be traced. In this way, an entire pangenome can be represented as a coloured *de Bruijn* graph, allowing for the identification of sequences, which are common or unique among the genomes that constitute the pangenome [22,23]. In the iterative mapping and assembly approach, a single whole genome assembly is used as the basis of the pangenome. Reads from other individuals are mapped one at a time to this reference, and the unmapped reads are extracted and assembled. The reference is updated with the newly assembled sequence, and this updated reference is used to sequentially map reads from other individuals. This approach is particularly useful when low coverage samples are available.

In the last five years or so, pangenomic studies in plants have become popular. A number of examples are available in a range of species such as maize [8,9], soybean [10,11], *Brassica rapa* [15], *Brassica oleracea* [14], and rice [12,13]. Irrespective of the species under study, the main aims have been to gain a better understanding of the core and variable genomes, and to identify candidate genes, which are associated with phenotypic variation. Analysis of 27 maize lines showed that the reference genome B73 only represented 70% of the entire pangenome [9]. Another study involving 503 diverse maize inbred lines revealed that 8681 representative transcript assemblies were not present in the B73 reference genome [8]. During the de novo assembly of seven diverse accessions of *Glycine soja* and comparative genomics analysis of these accessions with cultivated soybean (*G. max*), it was found that 80% of the pangenome was present in all accessions while the remainder was variable and displayed higher sequence variation compared to the core genome [10]. In another study involving 17 wild and 14 cultivated soybean genomes, it was found that higher genetic variation was present in wild soybean accessions [11]. During comparison and functional annotation of three *Brassica rapa* genomes (a turnip, a rapid cycling, and a Chinese cabbage), it was revealed that a significant amount of divergence had already occurred between *B. rapa* genotypes prior to domestication [15]. In a more recent study revolving around the *B. oleracea* pangenome, it was found that almost 20% of the genes were affected by presence/absence variation [14]. In a pangenome study of three divergent rice lines, it was revealed that 92% of the genes were core genes, while the remaining were variable, shorter in length, and had fewer exons [12]. Yet another study involving 1483 cultivated rice accessions showed that protein-coding genes not present in the Nipponbare reference genome had been successfully assembled [13].

## 2. Single Nucleotide Polymorphisms

Single nucleotide polymorphisms (SNPs) are single base pair positions in genomic DNA at which alternative alleles occur [24,25]. SNPs can be divided into transversions (C/G, A/T, C/A, and T/G) and transitions (C/T or G/A). The majority of SNPs at any given site are bi-allelic, but tri-allelic and tetra-allelic SNPs also exist [25]. The advent of next-generation sequencing and the development of high-throughput methods for their detection have revolutionized the use of SNPs as molecular markers. SNPs have become the marker of choice in genetic analysis of human, animal, microbial, and plant data. In human genetics for instance, SNPs are being used for the detection of alleles linked to genetic diseases. SNPs can be used to generate very high-density genetic maps, which can be used to develop haplotyping systems for candidate genes/regions. In addition, the low mutation rate of SNPs makes them ideal for studying complex genetic traits and understanding genome evolution [26].

Before the advent of next-generation sequencing, methods for SNP detection were relatively low-throughput and did not require prior sequence knowledge. SNPs were detected using DNA conformational changes or recognition of restriction enzyme site differences [25]. However, in the last decade or so, the detection of SNPs started to occur in a high-throughput manner due to the increase in sequencing capacity, resulting in longer reads and greater overall sequence output [25,27]. SNP calling usually starts with read mapping, followed by processing of the mapped reads, variant calling, and finally variant filtering. Depending on the quality of the sequence data, pre-processing of reads may be required prior to mapping. This step should not be overlooked as it increases the chance that a read will align to the reference and it reduces the likelihood that sequencing errors will be mistaken for SNPs [27]. There are numerous next-generation sequencing (NGS) alignment tools available today, each with their own specificities. Some of the most widely used aligners for read mapping include Bowtie2 [28], BWA [29], and SOAP2 [30]. Read mapping is followed by processing of the mapped reads; this usually involves marking/removal of duplicates. Picard MarkDuplicates (http://broadinstitute.github.io/picard) marks duplicate reads without removing them. It performs additional tasks such as estimating the percentage of optical duplicates, which are sequences that arise from one flow cell cluster, but are incorrectly identified as being part of multiple adjacent clusters. These are different from PCR duplicates, which occur as a result of the same DNA molecule being present in two different flow cell clusters after PCR amplification. Post-alignment processing may also involve removal of reads that fall below a given mapping quality threshold or reads that do not map in concordant pairs. Post-alignment processing is followed by variant calling. The latter process can be carried out using heuristic-based or probability-based algorithms. SNP callers using a heuristic approach rely on the abundance and quality of data. Two such SNP callers are VarScan2 [31] and SGSautoSNP [32]. VarScan2 combines read depth, base quality, and variant allele frequency with Fisher’s exact test to compare the number of reads supporting each allele with the expected distribution based solely on sequencing error. SGSautoSNP relies on a redundancy approach for accurate SNP calling. One of most notable features of this pipeline is that it only uses the reference to position reads from different individuals and finds variants between the mapped reads. This reduces the bias that may result from errors in genome assemblies. As far as probability-based algorithms are concerned, they make use of Bayes’ theorem to call SNPs. The underlying principle is that the probability of the observed genotype being the true genotype depends on the prior possibilities of each possible genotype, and the probability distribution of the data given each possible genotype [33]. Probability-based algorithms can further be divided into two groups: haplotype-based callers and single site-based callers. In the former group, haplotypes are computed either by read mapping, local read assembly, or a combination of both. Such callers include HaplotypeCaller [34,35,36], and Platypus [37]. Single site-based callers, as the name suggests, consider each site individually. Examples of such tools are Samtools/Bcftools [38], SOAPsnp [39], and UnifiedGenotyper [34,35,36].

SNPs can be identified at the genome-wide level. Genomic SNPs tend to be evolutionarily neutral, meaning they are not subject to selective pressures; their abundance in a population depends on random genetic drift, allowing for a more complete estimate of diversity levels [40]. SNPs can also occur in genes. These SNPs can be synonymous or non-synonymous. The former do not cause a change in the amino acid being translated, but the latter result in a different amino acid being translated. Non-synonymous SNPs within a transcribed gene can alter its protein structure or function, thereby affecting an organism’s development or response to environment. While genic SNPs can be used for whole genome scanning of linkage disequilibrium (LD) for trait dissection and gene mapping in crops for instance [24], the use of such SNPs alone can lead to an underestimation of true SNP number due to evolutionary constraints, thereby providing reduced resolution for genetic diversity studies. In addition, genic SNPs can increase the number of false SNPs being identified, especially in polyploid species, since expressed sequence data that originate from different homeologous and paralogous loci cannot be easily differentiated among inter-varietal sequences [25].

## 3. How Can the Availability of a Pangenome Increase the Efficiency of SNP Discovery?

The majority of studies to date have relied on a single reference genome to call SNPs between multiple individuals. However, using a pangenome, which represents the complete gene content of any given species, would increase the efficiency of SNP calling in several ways. Irrespective of the algorithm used, or the approach (reference-based or reference-free) employed to identify SNPs, using the pangenome as the reference for read mapping would take into account regions displaying PAVs. In this way, it will be possible to know the number and types of SNPs contributed by each individual in the pangenome. This would also increase the overall number of SNPs that would be identified if the reference had otherwise been based on a single individual alone. In addition, using the pangenome as a basis for SNP discovery would cut down the time and effort required to map reads to several individuals one at a time. This also means that SNP results coming from different references would not have to be consolidated into a single SNP set after analysis. Another major advantage of using the pangenome for SNP discovery is that it allows for the discrimination of SNPs, which occur in core and variable regions of the pangenome. Identification of SNPs in the variable genome for instance can be helpful in characterizing novel metabolic pathways [40] and finding molecular fingerprinting targets for use in epidemiological and population genetics studies [41]. A number of software packages which have been designed for the analysis of pangenomes in mind, can assist with this task. Panseq [42] is one such tool. Among other functions, this online program is able to identify core and variable SNPs, and has a locus selector module that is able to select the most discriminatory loci among the variable loci or core gene SNPs.

## 4. Applications of Discovered SNPs in Relation to Pangenomes

Various applications of SNP markers exist. SNPs have been used extensively in plants for the purpose of crop improvement. Some of the applications include studying genetic diversity, constructing high-resolution genetic maps, LD-based association mapping, and phylogenetic analysis [43]. Information on genetic diversity and relationships among crop varieties is of great importance for germplasm conservation, assignment to heterotic groups and inbred line identification. This information can also assist with the identification of novel alleles, which can be introgressed into elite lines [44]. When using a pangenome as the basis for SNP discovery, SNPs may be identified in regions showing PAV, and in this way it will be possible to identify which variety and its contributing SNP can be used for the introgression of novel alleles. This is especially useful for crops, which as a result of domestication breeding, have lower genetic diversity compared to their wild relatives [45,46].

The abundance of SNPs, coupled with high-throughput discovery and detection methods make them ideal candidates for use in genetic studies involving linkage mapping, map-based positional cloning, and quantitative trait loci (QTL) mapping [44]. In the case of pangenomes, the identification of rare variants associated with QTLs for agronomic traits can help with the improvement of cultivars through breeding. For instance, during the analysis of the pangenome of *Glycine soja*, which is the wild relative of the soybean *Glycine max*, the majority of genes affected by SNPs or indels causing stop codon gain or loss and frameshift were rare events, usually found in only one of the seven *G. soja* accessions [10]. As an example, the gene *Glyma02g25230*, which is one of the two homologs of *Spiral2*, a key microtubule gene for directional cell elongation that is associated with the right-handed helical growth in *Arabidopsis* [47], was found to harbour three indels in all *G. soja* accessions, but not in *G. max.* These indels were responsible for amino acid changes in one of the Huntingtin, elongation factor 3 (EF3), protein phosphatase 2A (PP2A), and the yeast kinase TOR1 (HEAT)-repeat motifs, suggesting the potential association with the twining growth habit exhibited by *G. soja* compared to the erect growth found in *G. max.* This study also highlighted the potential of using newly identified genetic variation contained within genomic regions that have been fixed in *G. max.* This information may be used to design crosses to determine if the fixed regions are associated with phenotypes of agricultural value, thereby providing additional candidate genes for the development of new, improved varieties.

SNPs, if associated with a target trait, can be used for marker-assisted selection (MAS) to identify individuals containing a combination of alleles of interest from large segregating populations [44]. SNPs can be identified within or close to genes associated with agronomic traits. Although the SNPs may not directly be responsible for the mutant phenotype, they may be applied for MAS and for the positional cloning of the gene in question [48]. This can be achieved via the development of haplotyping systems for candidate genes/regions in the genome. The information provided by SNPs is useful when several SNPs define haplotypes in candidate regions. Ideally, a subset of SNPs, which are deemed to be informative enough to perform association studies but still small enough to reduce the analysis workload would be selected [49]. This approach is known as representative SNP selection and it reduces the amount of redundancy when studying parts of a genome associated with traits [50]. In the case of pangenomes, the identification of additional SNPs, which would not have been possible using a single reference alone, could contribute to the identification of novel haplotype blocks and their representative SNPs, which could be used for MAS. For instance, during the construction of the rice pangenome using 1483 cultivated accessions, association mapping was performed for grain width and 840 metabolic traits using SNPs identified on the dispensible genome. It was found that 41.6% of trait-associated SNPs in general were found on the variable genome, and that 23.5% of metabolic traits had higher associations with SNPs on the variable genome compared to the core genome [13].

Phylogenetic studies in plants have traditionally relied on sequence diversity in genes of interest. SNPs in nuclear and chloroplast genes represent a rich source of phylogenetic information that has been used to elucidate the evolutionary relationships in a wide variety of crop species. The analysis of SNP diversity and conservation between the sequences of different individuals can help better understand patterns of inheritance. By considering rates of mutation, a molecular clock may also be applied to estimate the timing of species divergence. Molecular phylogenetics has been applied to a number of plant genomes [44]. One example is the study of maize genome evolution. The order and timing of waves of historical transposon activity has been made possible through a comparison of the terminal inverted repeats of transposons in regions of the maize genome [51].

The advantage of using pangenomes for phylogenetic analysis is that with the additional information provided by SNPs identified in regions showing PAV, it will be possible to infer more accurate relationships between accessions. Furthermore, the variable genes can be used to identify which genes are uniquely present and absent in each accession, and these numbers can be placed on the phylogenetic tree. In the recent publication by Golicz et al. [14], the placement of the number of variable genes on the phylogenetic tree of the *B. oleracea* pangenome allowed for the visualization of not only the number of uniquely present and absent genes for each accession, but also the number of genes present and absent in different combinations of accessions. In addition, the length of each branch in the tree was proportional to the number of nucleotide substitutions per site. The evolutionary patterns observed in the tree can then be linked to agronomic traits associated with each accession.

Advances in NGS and bioinformatics software have allowed SNPs to be readily available, and in large numbers. This has in turn allowed the identification of genomic regions that show evidence of selective pressure, thereby helping us understand how populations and species evolve [52,53]. In the case of plants, these signatures of selection have been shown to be associated with regions of the genome that are associated with traits of interest as indicated by a number of studies in crop species such as soybean [54], wheat [55], rice [56], and maize [57]. Various methods for detecting evidence of selection exist, and they all revolve around SNPs. One widely used example is the F_ST_ statistic, which uses differences in allele frequency between populations to determine the presence of selective pressure in one population compared to another [58]. Another statistic is Tajima’s D, which can be used to detect selective sweeps, which lead to an increase in frequency of alleles that confer a selective advantage [59]. Yet another way to evaluate selective pressures in genic regions is to compare the rate of non-synonymous amino acid substitutions per non-synonymous sites to the rate of synonymous substitutions per synonymous sites (Ka/Ks) [60]. Under neutral selection, Ka/Ks = 1. On the other hand, Ka/Ks > 1 and Ka/Ks < 1 indicate negative (purifying) selection, and a positive (adaptive) selection, respectively [61]. Since signatures of selection are surveyed in protein-coding genes, and the pangenome represents the full complement of genes in a given species, using a pangenomics approach to identify genes that have undergone positive or negative selection would yield more comprehensive results with respect to the species under study. For instance, during the construction of the soybean pangenome, intergenomic comparisons of *G. max* with seven wild accessions of *G. soja* identified 682 genes in *G. max* involved in abiotic stress regulation, which were found to have undergone positive selection [10]. On the other hand, the *G. soja* accessions had fewer genes (ten positively-selected genes shared by at least three wild accessions) that were under positive selection, implying that adaptation to different environments may have been the driving force behind this phenomenon. This study also showed that the variable genome of the soybean pangenome had undergone weaker purifying selection and/or greater positive selection compared to core genes, suggesting that lineage-specific genes evolve faster than shared genes.

SNPs identified in the pangenome can also be used to place newly assembled contigs in the pangenome reference. This method is particularly useful when using the iterative mapping and assembly approach, which can result in a large number of contigs. Since the starting point of the assembly is a single reference, and new contigs are generated at each step of the iterative assembly process, it is very useful to know the positions and orientations of the contigs relative to the pangenome reference, especially if they harbour genic sequences. In studies involving association mapping or QTL analysis, candidate genes are mapped to a narrow genomic region in which the causal genes are selected based on the gene annotation of the reference genome [12]. In the case of pangenomes, if the positions of novel genes are not known, then such attempts would be futile. In a recent study involving the construction of a pangenome using low-coverage sequencing reads of 1483 cultivated *Oryza sativa* (rice) accessions, LD was used to determine the chromosome positions of each newly assembled contig relative to the Nipponbare reference genome. Reads from all accessions were aligned to the variable genome and SNPs were called between the accessions. The LD between a specific SNP identified against the new contigs and all the SNPs previously identified against the core genome, i.e., the Nipponbare reference, was calculated. The chromosome location of the SNP in the core genome having the highest LD with the SNP on a specific contig of the variable genome was deemed to be the approximate location of that contig. In this way, the genes found in the novel contigs were assigned to their correct genomic positions relative to the Nipponbare reference [13].

## 5. Challenges and Future Directions

The use of pangenomes for SNP discovery can pose some challenges. Similar to single linear reference genomes, the quality of pangenome assemblies in terms of completeness and annotation is of utmost importance. Mis-assembled genomes can impede the accurate alignments of reads, which can in turn affect downstream analyses such as the identification of large or small variants such as SNPs. Large, complex genomes such as many plant genomes are prone to mis-assemblies due to their repetitive nature [62]. Incorrectly assembled contigs/scaffolds can be misconstrued for structural variants, and if read mapping occurs in these regions, SNPs will be erroneously called. Similarly, if reads do not align where they should, the number of SNPs will be over or underestimated. Fragmented assemblies can also be produced as a result of repeats not being resolved during the assembly process [63,64], and the outcomes with respect to SNP discovery will be the same as for mis-assembled genomes. The quality of the gene annotation will also impact the functional annotation of SNPs in genic regions of the pangenome. Both mis-assembled and fragmented genomes can reduce the number of annotated genes, or give rise to partial genes, which can underestimate the number of SNPs. This can be problematic when evaluating signatures of selective pressures in genic regions. However, with the advent of long read-sequencing, reads that span several kilobases can be produced. Additionally, since the long reads can span complex and repetitive genomic regions, they will be able to resolve repeats during the assembly process [65]. Tools are actively being developed to handle the assembly of long reads with the promise of delivering high-quality genomes [66], and these should encourage more pangenomic studies.

Existing pangenome analysis tools have been designed with small microbial genomes in mind. However, with the increasing interest in pangenomic analyses of higher, more complex organisms such as plant genomes, there is a need to develop tools, which will be able to handle the deluge of information that will be produced. Tens of thousands of genes will have to be analysed. An even more considerable number of SNPs will be identified both in genic and non-genic regions. Therefore, there is a need for tools that will be able to assist with a number of tasks such as the detection of orthologous genes, pangenome modelling, functional annotation of SNPs and phylogenetic analyses, in both an accurate and timely manner. In addition, the results derived from the pangenomic analyses will have to be stored and maintained in large repositories, as well as allow fast access to users. The integration of genomic, gene expression data and SNP data will also be necessary to link the core genome and the variable genome with expression levels and variants such as SNPs and indels. Such functional information should be able to provide a link between the pangenome and the trait observed, and be made readily accessible to plant breeders.

## 6. Conclusions

It is becoming clear that the analysis of a pangenome rather than a single linear reference genome will ensure that the entire genetic diversity within any given species is fully represented. The use of long read sequencing technologies coupled with the reduced cost associated with NGS will make high quality genome assemblies more readily available to the research community, and this will fuel more pangenome studies in the future. The additional information captured within the pangenome in the form of variation at both the gene level and the nucleotide level will provide a valuable link between genotypic data and phenotypic data, especially in the case of crop genomics. With the onset of climate change, crops, which are able to adapt to a wide range of changing environments, are being highly sought after. Therefore, the evolution of variable genes and their associated SNPs can greatly help with the improvement of crop varieties in the light of an increasing global population and an ever-changing climate.
